# A Randomized Controlled Trial to Compare the Role of Intravenous Iron Sucrose Versus Oral Ferrous Ascorbate for the Prophylaxis of Anemia in Pregnant Women

**DOI:** 10.7759/cureus.82941

**Published:** 2025-04-24

**Authors:** Maganti Yamini Purna, Neelamma Patil, Rajasri G Yaliwal, Aruna Biradar, Shobha Shiragur

**Affiliations:** 1 Obstetrics and Gynaecology, Shri BM Patil Medical College Hospital & Research Centre, Vijayapura, IND

**Keywords:** ferrous ascorbate, iron deficiency anemia, iron sucrose, pregnancy, prophylaxis

## Abstract

Background and objective

Anemia in pregnancy is a significant health concern, particularly in low- and middle-income countries such as India. Iron deficiency anemia (IDA) is its most common cause, leading to adverse maternal and fetal outcomes. While oral iron (OI) therapy is the standard prophylaxis, poor compliance due to gastrointestinal side effects limits its effectiveness. Intravenous iron sucrose (IVIS) may provide an alternative with better tolerability. This study aimed to compare the efficacy, safety, and compliance of IVIS versus OI for anemia prophylaxis in pregnant women.

Materials and methods

A randomized controlled trial was conducted at Shri BM. Patil Medical College, Hospital and Research Centre, Vijayapura, Karnataka, over 12 months. A total of 100 antenatal women with hemoglobin (Hb) ≥11 g/dL were randomized into two groups: Group A received IVIS 200 mg in 100 mL normal saline at 20-24, 24-28, and 28-32 weeks, and Group B received OI 100 mg daily till 32 weeks. Hematological parameters and serum ferritin were measured at baseline, four weeks, and 12 weeks of treatment. Side effects, compliance, and cost of the treatment were assessed.

Results

Group A showed a significantly greater increase in Hb (12.19 ± 0.68 g/dL) compared to Group B (11.63 ± 0.74 g/dL) at 12 weeks (p<0.0001). Serum ferritin levels were significantly higher in Group A (163.26 ± 76.28 ng/mL) versus Group B (81.28 ± 65.98 ng/mL) (p<0.0001). Gastrointestinal side effects were more common in the oral group. Notably, the cost of treatment for Group A was also lower

Conclusions

Based on our findings, IVIS is more effective, better tolerated, and improves iron stores significantly compared to OI for the prophylaxis of anemia in pregnancy.

## Introduction

Anemia during pregnancy is a significant global health issue, affecting a large proportion of pregnant women, particularly in low and middle-income countries. According to the World Health Organization (WHO), anemia in pregnancy is defined as a hemoglobin (Hb) concentration below 11 g/dL, and it can be classified into mild, moderate, and severe categories based on Hb levels. The prevalence of anemia in pregnancy is alarmingly high, with the WHO estimating that approximately 37% of pregnant women are affected [1]. It is a critical concern due to its association with poor maternal and fetal outcomes, including increased maternal mortality, preterm births, low birth weight (LBW), intrauterine growth restriction (IUGR), and perinatal morbidity and mortality [2]. In India, anemia is responsible for about 40% of maternal deaths, either directly or indirectly, and when Hb levels fall below 5 g/dL, maternal mortality increases significantly [3].

Iron deficiency anemia (IDA) is the leading cause of anemia during pregnancy, due to increased iron requirements during pregnancy that often exceed the available iron stores. The recommended iron intake for pregnant women increases from 0.8 mg/day in early pregnancy to 7.5 mg/day in the later stages [4]. Despite various governmental initiatives, such as the National Nutritional Anemia Control Programme (NNACP), which advocates for daily iron supplementation during pregnancy, anemia continues to be prevalent among pregnant women in India [5]. The standard practice under the NNACP includes the administration of oral iron (OI) tablets containing 60 mg of elemental iron and 500 mcg of folic acid. However, many pregnant women do not adhere to the regimen due to gastrointestinal side effects, poor tolerance, and inadequate efficacy [6].

Iron therapy, both oral and intravenous, remains the cornerstone of the treatment for IDA. OI, despite being cost-effective and accessible, often leads to gastrointestinal side effects like nausea, constipation, and abdominal discomfort, which may reduce patient adherence. Hence, intravenous iron formulations have been increasingly utilized as an alternative to overcome the limitations of OI. Intravenous iron sucrose (IVIS) is a widely used parenteral form of iron therapy that has shown promise in treating anemia, particularly in cases where oral iron therapy is not tolerated or effective [7]. This treatment offers the advantage of bypassing the gastrointestinal tract, thereby avoiding the common adverse effects of OI and potentially improving compliance and outcomes [8]. However, its higher cost compared to OI raises concerns regarding its widespread use, especially in resource-limited settings.

Anemia during pregnancy remains a major public health concern in India, significantly affecting both maternal and fetal outcomes. Effective prophylactic strategies are essential to address this burden and improve maternal health. This study seeks to compare the efficacy of IVIS and OI in preventing anemia among pregnant women. By evaluating improvements in Hb levels, treatment compliance, safety profiles, and cost-effectiveness, the study aims to identify the optimal approach for clinical practitioners. As a randomized controlled trial, it also aims to provide evidence on the impact of these interventions on iron stores and overall maternal well-being. This study aims to compare the efficacy, safety, compliance, and cost-effectiveness of IVIS versus oral ferrous ascorbate in the prophylaxis of anemia in pregnant women.

## Materials and methods

This randomized controlled trial was conducted to compare the effectiveness of prophylactic IVIS versus OI in pregnant women. The study was carried out at Shri BM. Patil Medical College, Hospital and Research Centre, Vijayapura, Karnataka, over 12 months: from April 2024 to March 2025. The study population consisted of antenatal women with a confirmed intrauterine pregnancy, attending the Obstetrics and Gynecology outpatient department. A total of 100 participants were included in the study, with 50 women assigned to each treatment group through randomization. Sample size calculation was performed using G Power 3.1.9.4 software, which indicated a required sample size of 80 participants (40 in each group) to achieve 92% power at a 5% significance level. Accounting for a 20% dropout rate, the final sample size was adjusted to 100 participants, with 50 women in each group.

The inclusion criteria were as follows: a Hb level of ≥11 g/dl, a singleton pregnancy, and the ability to provide written informed consent. Women with a history of hemoglobinopathies, hypertensive disorders of pregnancy, diabetes mellitus, chronic bleeding, heart diseases, or multiple pregnancies were excluded from the study. We obtained approval from the Institutional Ethics Committee (IEC approval no: BLDE (DU)/IEC/959/2022-23, dated 10/4/2023) and received informed consent from participants. All antenatal women underwent a general physical examination, obstetric evaluation, and routine blood investigations before randomization into the two groups. Randomization was performed using a computer-generated random number table. Allocation concealment was ensured using sealed, opaque, sequentially numbered envelopes, and group assignment was carried out by a research assistant not involved in outcome assessment. Group A was assigned to receive IVIS, while Group B received oral ferrous ascorbate (OI) supplementation.

In Group A, participants were administered IVIS 200 mg in 100 ml of normal saline, which was infused over 15-20 minutes at 20-24 weeks, 24-28 weeks, and 28-32 weeks of gestation. In Group B, participants received OI, providing 100 mg of elemental iron daily at bedtime, one hour before meals, till 32 weeks. Hb levels, RBC count, packed cell volume (PCV), mean corpuscular volume (MCV), mean corpuscular hemoglobin (MCH), mean corpuscular hemoglobin concentration (MCHC), and serum ferritin levels were assessed before starting prophylactic treatment and then at four weeks and 12 weeks after treatment initiation. Side effects such as gastrointestinal symptoms (nausea, vomiting, epigastric pain, constipation, diarrhoea), rashes, itching, chills, headache, and local pain at the injection site were carefully monitored and recorded. The trial was registered prospectively with the Clinical Trials Registry - India (CTRI) under the registration number CTRI/2024/04/066003. The study adhered to the principles of the Declaration of Helsinki and Good Clinical Practice guidelines. This randomized controlled trial has been reported in accordance with the CONSORT (Consolidated Standards of Reporting Trials) guidelines [9].

**Figure 1:**
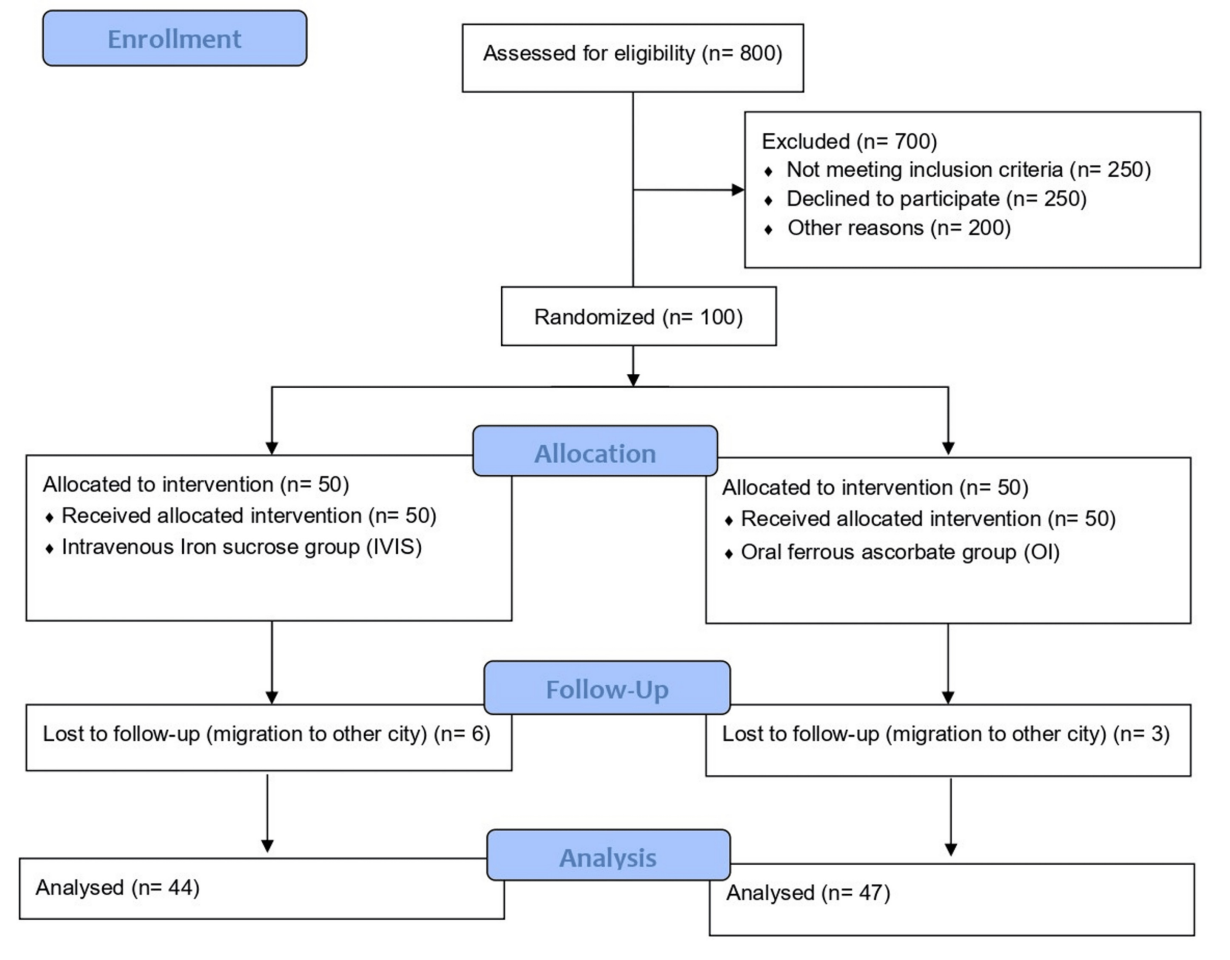
CONSORT flow diagram depicting the selection of studies CONSORT: Consolidated Standards of Reporting Trials

The primary outcome of the study was to assess the efficacy of IVIS in maintaining Hb levels compared to OI during pregnancy. Secondary outcomes included evaluating the compliance, iron stores, and cost-effectiveness of parenteral versus OI supplementation, as well as the safety of both methods. Data were analyzed using Microsoft Excel and SPSS Statistics software version 26.0 (IBM Corp., Armonk, NY). Descriptive statistics, including mean and standard deviation (SD), were used for continuous variables, while categorical variables were analyzed using the chi-square test or Fisher’s exact test. The difference between continuous variables was assessed using the z-test, and a p-value ≤0.05 was considered statistically significant (Figure 1)

## Results

There were no significant differences between the two groups regarding the demographic profile of participants: age, residence, and gravida status. The majority of the participants were aged between 21 and 25 years, accounting for 24 (54.5%) and 23 (48.9%) in Group A (IV IS) and Group B (OI), respectively. The majority of participants in both groups resided in rural areas: 29 (65.9%) in Group A and 30 (63.8%) in Group B. A similar distribution was observed for gravida status, with primigravida women making up 68.2% of Group A and 66% (n=31) of Group B. These findings suggest that both groups were demographically comparable, and no significant association was observed for these variables (Table 1).

**Table 1:** Demographic profile of participants A p-value less than 0.05 was considered significant IVIS: intravenous iron sucrose; NS: not significant; OI: oral iron

Variable	Group A (IVIS) (n=44), n (%)	Group B (OI) (n=47), n (%)	Total (N=91), n (%)	P-value (chi-squared test)
Age, years				
≤20	6 (13.6%)	8 (17%)	14 (15.4%)	0.89 (NS)
21-25	24 (54.5%)	23 (48.9%)	47 (51.6%)	
26-30	10 (22.7%)	10 (21.3%)	20 (22%)	
31-35	4 (9.2%)	6 (12.8%)	10 (11%)	
Residence				
Rural	29 (65.9%)	30 (63.8%)	59 (64.8%)	0.84 (NS)
Urban	15 (34.1%)	17 (36.2%)	32 (35.2%)	
Gravida				
Primigravida	30 (68.2%)	31 (66%)	61 (67%)	0.82 (NS)
Multigravida	14 (31.8%)	16 (34%)	30 (33%)	

Anemia was seen in three (6.8%) cases in Group A, and eight (17%) cases in Group B, and this association was non-significant (p=0.14). Hb levels significantly increased in Group A at both four weeks (p=0.005) and 12 weeks (p<0.0001), with this group showing a greater improvement compared to Group B, where only a modest improvement was observed after four weeks (p=0.005) and 12 weeks (p<0.0001). Regarding RBC count, no significant changes were noted at any time point between the two groups (p>0.05). PCV showed significant improvement in Group A at both four weeks (p=0.04) and 12 weeks (p<0.0001), while Group B showed a decrease in PCV at both time points. MCV, MCH, and MCHC did not exhibit significant differences between the two groups at any time point (p>0.05). Hence, while IVIS was more effective than OI in improving Hb and PCV levels, both treatments had similar effects on MCV, MCH, and MCHC over the 12 weeks (Table 2).

**Table 2:** Comparison of hematological parameters over time A p-value less than 0.05 was considered significant IVIS: intravenous iron sucrose; MCH: mean corpuscular hemoglobin; MCHC: mean corpuscular hemoglobin concentration; MCV: mean corpuscular volume; NS: not significant; OI: oral iron; PCV: packed cell volume; RBC: red blood cell count; S: significant; SD: standard deviation

Variables at different time points	Group A (IVIS) (n=44), mean ± SD	Group B (OI) (n=47), mean ± SD	P value (t-test)
Hemoglobin (g/dL)			
At baseline	11.81 ± 0.71	11.74 ± 0.86	0.67 (NS)
After 4 weeks	11.49 ± 0.84	10.99 ± 0.83	0.005 (S)
After 12 weeks	12.19 ± 0.68	11.63 ± 0.74	0.0001 (S)
RBC			
At baseline	4.12 ± 0.35	4.11 ± 0.41	0.85 (NS)
After 4 weeks	4.25 ± 0.40	3.87 ± 0.44	0.12 (NS)
After 12 weeks	5.14 ± 0.84	4.01 ± 0.42	0.19 (NS)
PCV (%)			
At baseline	34.86 ± 2.59	35.30 ± 2.79	0.42 (NS)
After 4 weeks	34.97 ± 2.83	33.39 ± 2.62	0.04 (S)
After 12 weeks	36.12 ± 3.46	33.60 ± 2.51	0.0001 (S)
MCV			
At baseline	84.70 ± 6.02	86.35 ± 5.78	0.17 (NS)
After 4 weeks	85.86 ± 5.94	87.40 ± 7.36	0.28 (NS)
After 12 weeks	86.80 ± 6.42	84.98 ± 6.99	0.21 (NS)
MCH			
At Baseline	28.56 ± 2.48	28.55 ± 2.19	0.98 (NS)
After 4 Weeks	28.71 ± 2.28	28.93 ± 3.03	0.71 (NS)
After 12 Weeks	29.26 ± 2.75	27.94 ± 2.73	0.02 (S)
MCHC			
At Baseline	33.60 ± 1.64	32.99 ± 1.90	0.09 (NS)
After 4 Weeks	33.40 ± 1.00	32.93 ± 1.44	0.07 (NS)
After 12 Weeks	33.69 ± 1.95	33.12 ± 1.91	0.16 (NS)

In terms of serum ferritin levels, Group A exhibited significantly better results compared to Group B. At baseline, serum ferritin levels were lower in Group A (51.90 ± 44.92 ng/mL) compared to Group B (63.33 ± 33.06 ng/mL), although this difference was not statistically significant (p=0.35). After 12 weeks, however, Group A's serum ferritin levels (163.26 ± 76.28 ng/mL) were significantly higher than those of Group B (81.28 ± 65.98 ng/mL) (p<0.0001), indicating that IVIS was more effective in increasing iron stores over time (Table 3).

**Table 3:** Comparison of serum ferritin levels A p-value less than 0.05 was considered significant IVIS: intravenous iron sucrose; NS: not significant; OI: oral iron; S: significant; SD: standard deviation

Time points	Group A (IVIS) (n=44), mean ± SD	Group B (OI) (n=47), mean ± SD	P-value (t-test)
At baseline	51.90 ± 44.92	63.33 ± 33.06	0.35 (NS)
After 4 weeks	98.36 ± 58.91	70.34 ± 44.75	0.09 (NS)
After 12 weeks	163.26 ± 76.28	81.28 ± 65.98	0.0001 (S)

**Figure 2:**
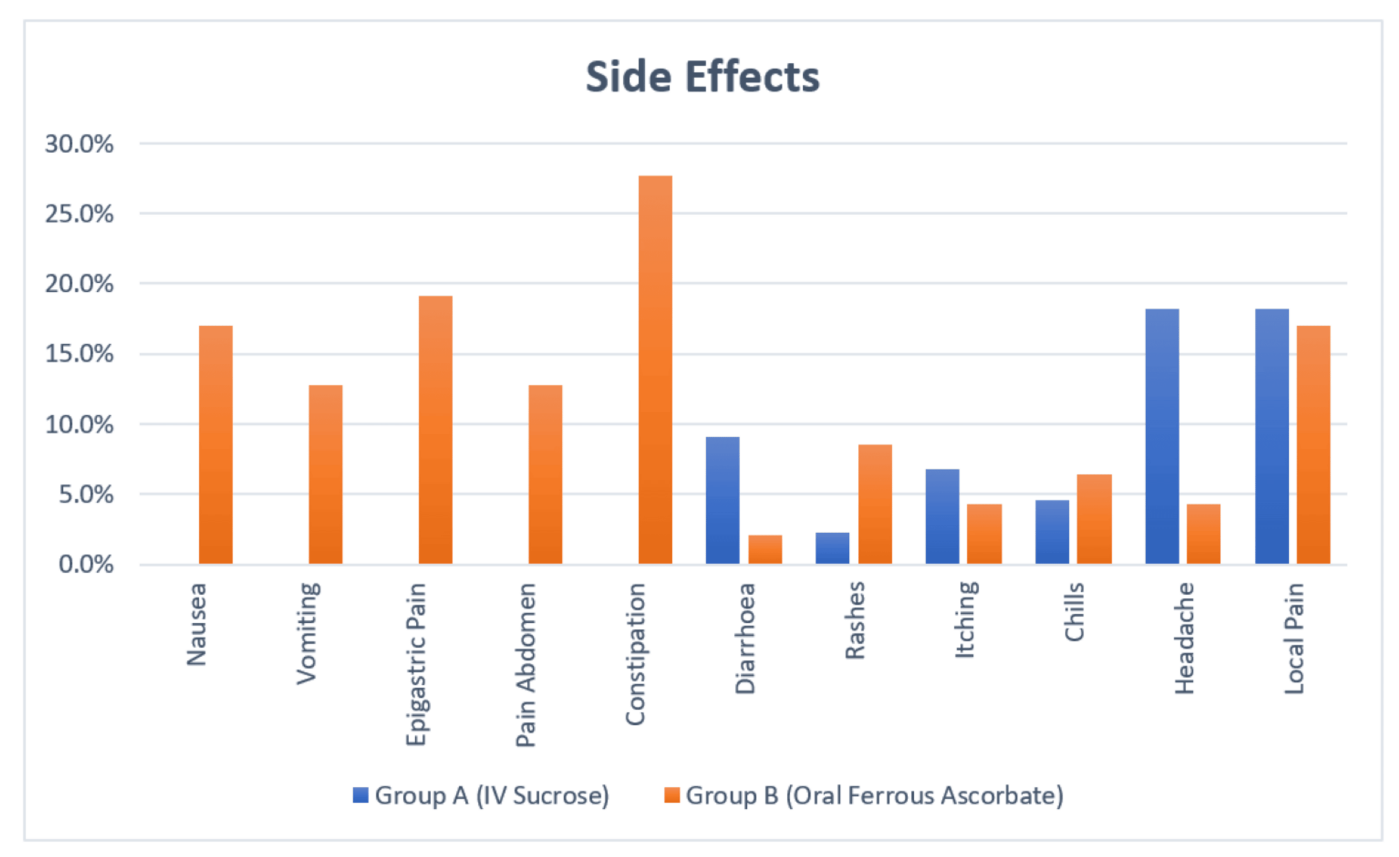
Comparison of side effects in both groups

Significant gastrointestinal side effects, including nausea, vomiting, epigastric pain, abdominal pain, and constipation, were more prevalent in Group B. In contrast, no such events were observed in Group A participants, but they exhibited higher incidences of rashes (n=4, 9.1%), chills (n=3, 6.8%), and local pain (n=8, 8.2%). There were no significant differences between the two groups regarding other side effects, such as itching, headache, and diarrhoea (Table 4, Figure 2).

**Table 4:** Frequency of side effects in the study groups A p-value less than 0.05 was considered significant IVIS: intravenous iron sucrose; NS: not significant; OI: oral iron; S: significant

Side effect	Group A (IVIS) (n=44), n (%)	Group B (OI) (n=47), n (%)	Total (N=91), n (%)	P-value (Fisher’s exact test)
Nausea	0 (0%)	8 (17%)	8 (8.8%)	0.003 (S)
Vomiting	0 (0%)	6 (12.8%)	6 (6.6%)	0.014 (S)
Epigastric pain	0 (0%)	9 (19.1%)	9 (9.9%)	0.002 (S)
Pain in the abdomen	0 (0%)	6 (12.8%)	6 (6.6%)	0.014 (S)
Constipation	0 (0%)	13 (27.7%)	13 (14.3%)	0.0001 (S)
Diarrhea	0 (0%)	1 (2.1%)	1 (1.1%)	0.33 (NS)
Rashes	4 (9.1%)	0 (0%)	4 (4.4%)	0.035 (S)
Itching	1 (2.3%)	1 (2.1%)	2 (2.2%)	0.96 (NS)
Chills	3 (6.8%)	0 (0%)	3 (3.3%)	0.067 (NS)
Headache	2 (4.5%)	0 (0%)	2 (2.2%)	0.14 (NS)
Local pain	8 (8.2%)	0 (0%)	8 (8.8%)	0.002 (S)

The follow-up data revealed that six (12%) participants in Group A (IVIS) were lost to follow-up, while only three (6%) in Group B (OI) were lost to follow-up. Overall, 9% of the participants were lost to follow-up across both groups. Regarding cost effectiveness, the mean cost of treatment in Group A (IVIS) was ₹2,152, while the mean cost in Group B (OI) was ₹2,464. The difference in costs between the two groups was statistically significant (p=0.03). This suggests that IVIS is more cost-effective compared to OI in the prophylaxis of anemia in pregnant women.

## Discussion

The age distribution of the participants in the current study shows that the majority of cases in both the IVIS group 24, 54.5%) and the OI group 23, 48.9%) belonged to the 21-25 year age group. The mean age of the participants in both groups was nearly identical, with the IVIS group having a mean of 24.62 ± 4.07 years and the OI group having a mean of 24.88 ± 3.86 years. This is consistent with findings from previous studies, such as those by Rudra et al. (25.08 ± 3.32 and 25.12 ± 3.73 years, respectively) and Sudakshina et al. (26 ± 4.7 and 24.7 ± 3.3 years, respectively) [10,11], indicating that both treatments are commonly applied to younger populations. Studies such as those by Dixit et al. and Savitha et al. also report similar age distributions, where the majority of cases fall within the reproductive age group [12,13].

In terms of gravida, the current research shows that 30 (68.2%) of cases in the IVIS group and 31 (66%) in the OI group were primigravida, with the remaining cases being multigravida. This contrasts with the findings of Rudra et al. [22 (22%) and 78 (78%) in IV and OI groups, respectively] and Savitha et al. [10 (33.7%) and 20 (66.7%) in IV and OI groups, respectively] [10,13]. The variation in gravida distribution between studies could also be influenced by differences in regional practices or population demographics.

The baseline Hb levels in the current study were 11.81 ± 0.71 in the IVIS group and 11.74 ± 0.86 in the OI group, which are similar to baseline levels reported by Rudra et al. and Sudakshina et al [10,11]. Over the course of the treatment, the IVIS group exhibited an increase in mean Hb from 11.81 ± 0.71 to 12.19± 0.68, whereas the OI group showed a decrease from 11.74 ± 0.86 to 11.63 ± 0.74. These findings differ from studies such as that by Thobbi et al., where the OI group showed a marked increase in Hb, suggesting that the treatment duration and frequency may have influenced the observed differences in Hb levels [14]. The IVIS group’s increase in Hb is consistent with studies by Agalya et al., who reported a more significant increase in Hb with IV supplementation compared to oral treatments, highlighting the quicker absorption and efficacy of IV iron [15]. The observed variations in outcomes between studies may reflect differences in treatment protocols, patient adherence, or the severity of anemia in the study population.

The mean PCV in the current study showed a significant increase in the IVIS group, from 34.86 ± 2.59 at baseline to 36.12 ± 3.46 at the end of treatment. In contrast, the OI group showed a decrease in PCV from 35.3 ± 2.79 to 33.6 ± 2.51. This pattern is consistent with the findings by Rudra et al. and Savitha et al., where a significant increase in PCV was observed in the IVIS group [10,13]. In terms of MCV, the current study found that while both groups showed slight increases in MCV, the differences between the groups were not statistically significant. This result contrasts with findings by Rudra et al., Sudakshina et al., and Savitha et al., where the IVIS group showed a more significant increase in MCV compared to the OI group [10,11,13]. The lack of significant change in MCV between the two groups in the current study could be due to the relatively short duration of treatment and variations in individual patient responses to iron supplementation.

The MCH in the current study showed minimal changes, with both groups exhibiting similar baseline values (28.56 ± 2.48 for IV and 28.55 ± 2.19 for oral) and slight increases by the end of the study. This is consistent with the study findings by Rudra et al., who reported modest changes in MCH following treatment [10]. Although MCH is a useful indicator of Hb content in red blood cells, the relatively stable values in the current study suggest that both IVIS and OI have a similar impact on this parameter.

Nausea, vomiting, epigastric pain, abdominal pain, and constipation were notably more prevalent in the OI group. Specifically, eight (17%) participants in the oral group experienced nausea, and six (12.8%) had vomiting, while none of the participants in the IVIS group reported these symptoms. This aligns with the findings of Thobbi et al, who also reported a higher incidence of nausea (n=6, 6%) and vomiting (n=3, 3%) in the oral group, while their IVIS group had no such side effects [14]. Similarly, Agalya et al. and Gogineni et al. observed higher rates of gastrointestinal disturbances in the OI group, with incidence rates of nausea and vomiting ranging from two (4%) to 15 (30%) [15,16].

Conversely, side effects such as rashes, chills, and local pain were more prevalent in the IVIS group in our study. Specifically, four (9.1%) participants in the IVIS group reported rashes, and eight (8.2%) experienced local pain at the injection site, while none of the participants in the OI group reported these effects. The study by Gogineni et al. also reported rashes in one (2%) patient in the IVIS group, but they found no rashes in the OI group, indicating a somewhat lower incidence compared to our findings [16]. Additionally, our study found a significant occurrence of chills (6.8%) in the IVIS group, which was not observed in the OI group, further emphasizing the specific side effects associated with IVIS.

In terms of efficacy, the current study showed that anemia was observed in three (6.8%) in the IVIS group and eight (17%) in the OI group, though this association was not statistically significant. This suggests that a higher proportion of antenatal women in the IVIS group were non-anemic compared to the OI group. Similar results were reported by Gogineni et al., where three (6%) participants in the IVIS group and nine (18%) in the oral group were anemic [16]. Regarding compliance, six (12%) participants in the IVIS group were lost to follow-up, while only three (6%) in the OI group were lost, contrasting with the study by Gogineni et al., where follow-up loss was significantly lower in the IVIS group (n=2, 4%) but higher in the OI group (40%) [16]. The current research found compliance rates of 44 (88%) in the IVIS group and 47 (94%) in the OI group, which is similar to the findings of Dixit et al., where the compliance rate was slightly higher in the IVIS group (93, 93%) compared to the OI group (92, 92%) [12].

The limitations of the study include a small sample size, which may limit the generalizability of the findings, and issues related to participant compliance, which could influence results. Additionally, the lack of sufficient studies on this topic highlights the need for further research. If more extensive research supports our findings, they can be considered standard practice in developing nations where anemia rates are higher and compliance is lower. The strengths of the study include its focus on comparing IVIS with ferrous ascorbate, offering a novel perspective. Furthermore, the measurement of key blood indices like mean Hb, RBC, MCV, MCH, MCHC, and serum ferritin strengthens the study's validity.

## Conclusions

This study aimed to evaluate the efficacy of IVIS versus OI for the prophylaxis of anemia during pregnancy. The results demonstrated significant improvements in Hb levels in the IVIS group compared to the OI group. Additionally, the IVIS group showed fewer side effects, making it a safer option. Notably, the cost of treatment for the IVIS group was also lower, making it a more cost-effective alternative. Based on these findings, IVIS is a more effective alternative for preventing anemia in pregnant women, with fewer side effects.
